# Supplementary figures and images for: Engineering an Exo70 integrated domain of a barley NLR for improved blast resistance
Source: Plant Cell. 2026 Jun 9;38(6):koag168. doi: 10.1093/plcell/koag168 (PMC13291817; doi:10.1093/plcell/koag168)

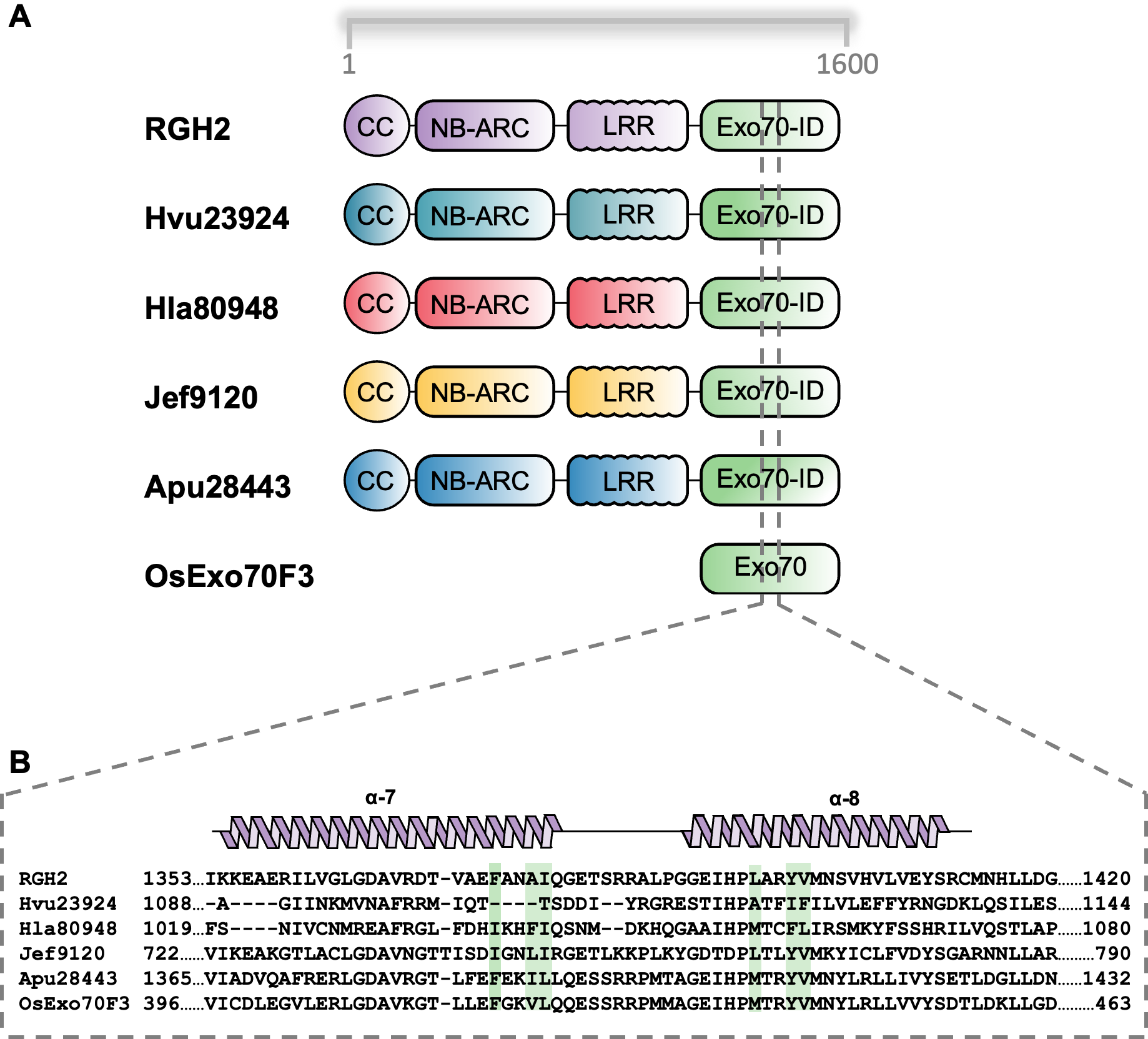

Supplement: koag168_Supplementary_Data [file koag168_supplementary_data.zip › Figure_S1.tiff]

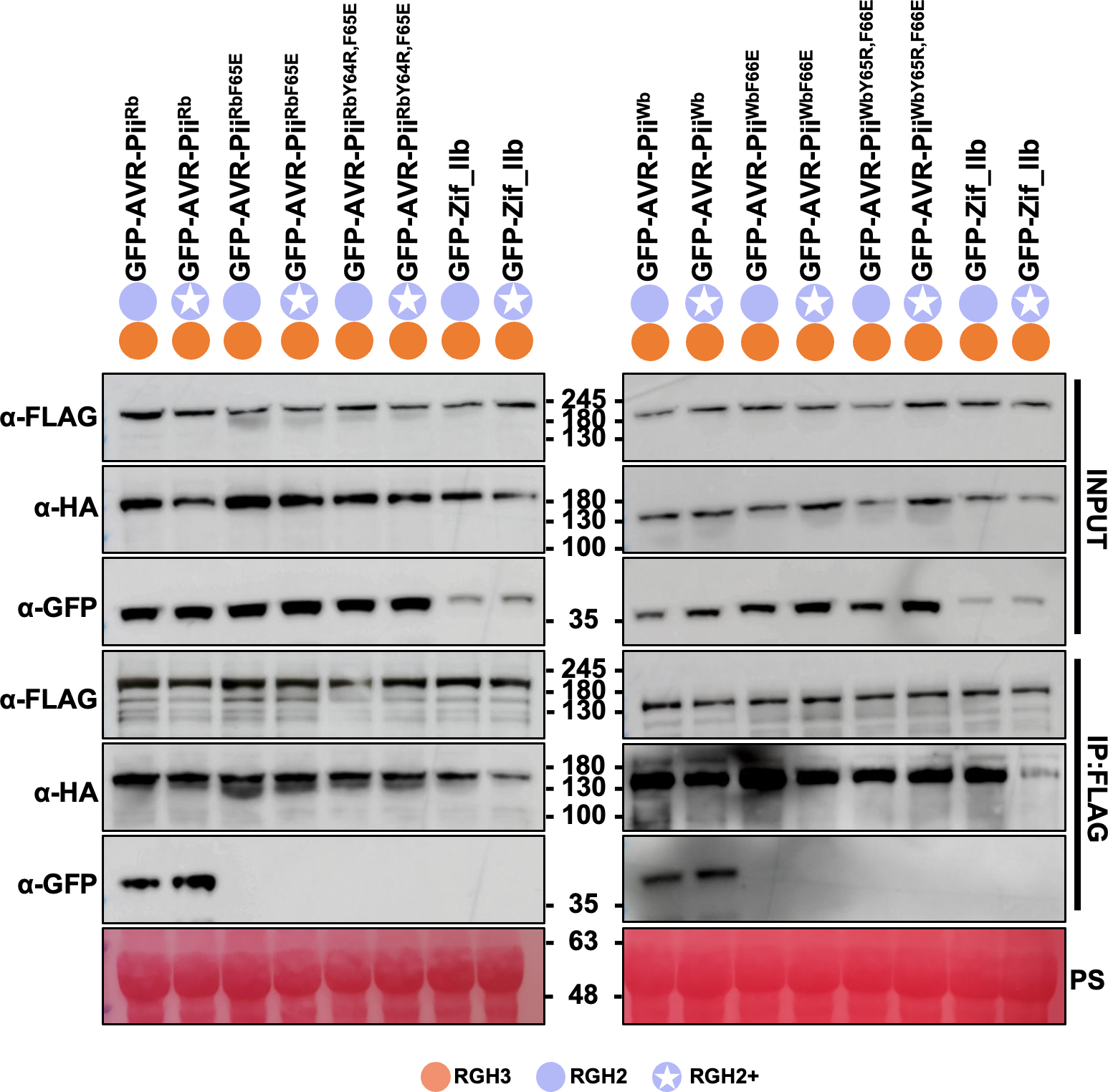

Supplement: koag168_Supplementary_Data [file koag168_supplementary_data.zip › Figure_S10.tiff]

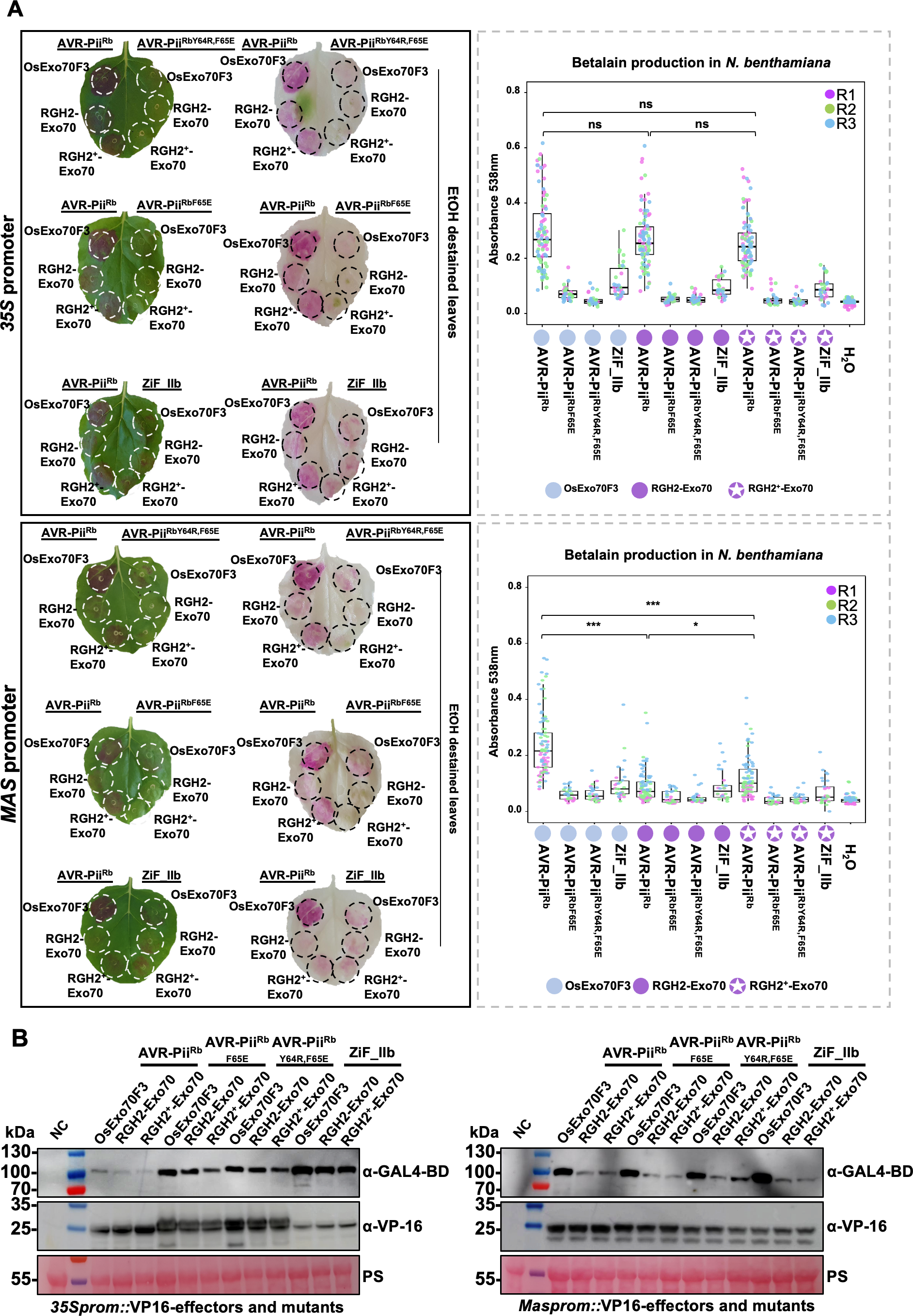

Supplement: koag168_Supplementary_Data [file koag168_supplementary_data.zip › Figure_S11.tiff]

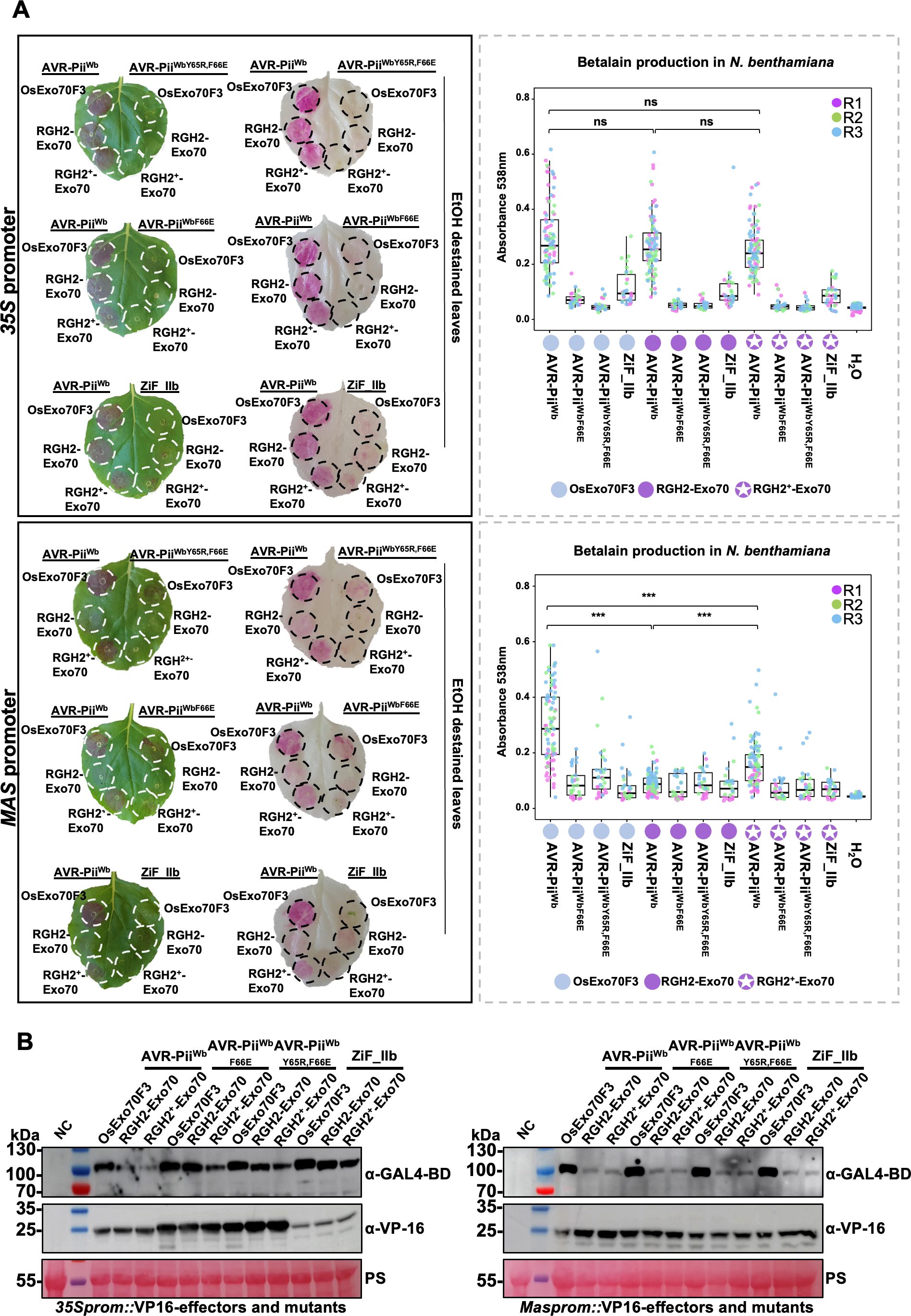

Supplement: koag168_Supplementary_Data [file koag168_supplementary_data.zip › Figure_S12.tiff]

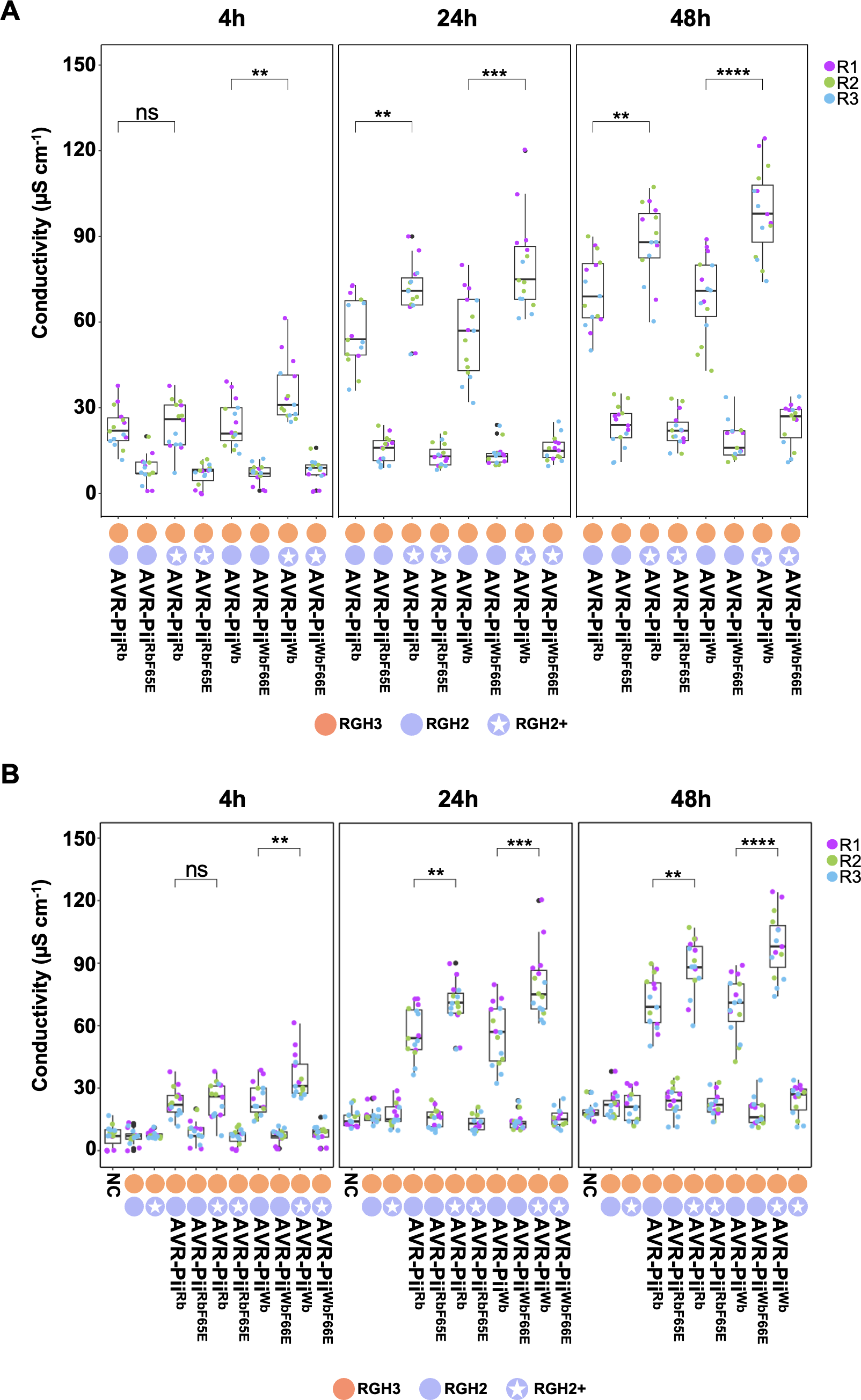

Supplement: koag168_Supplementary_Data [file koag168_supplementary_data.zip › Figure_S13.tiff]

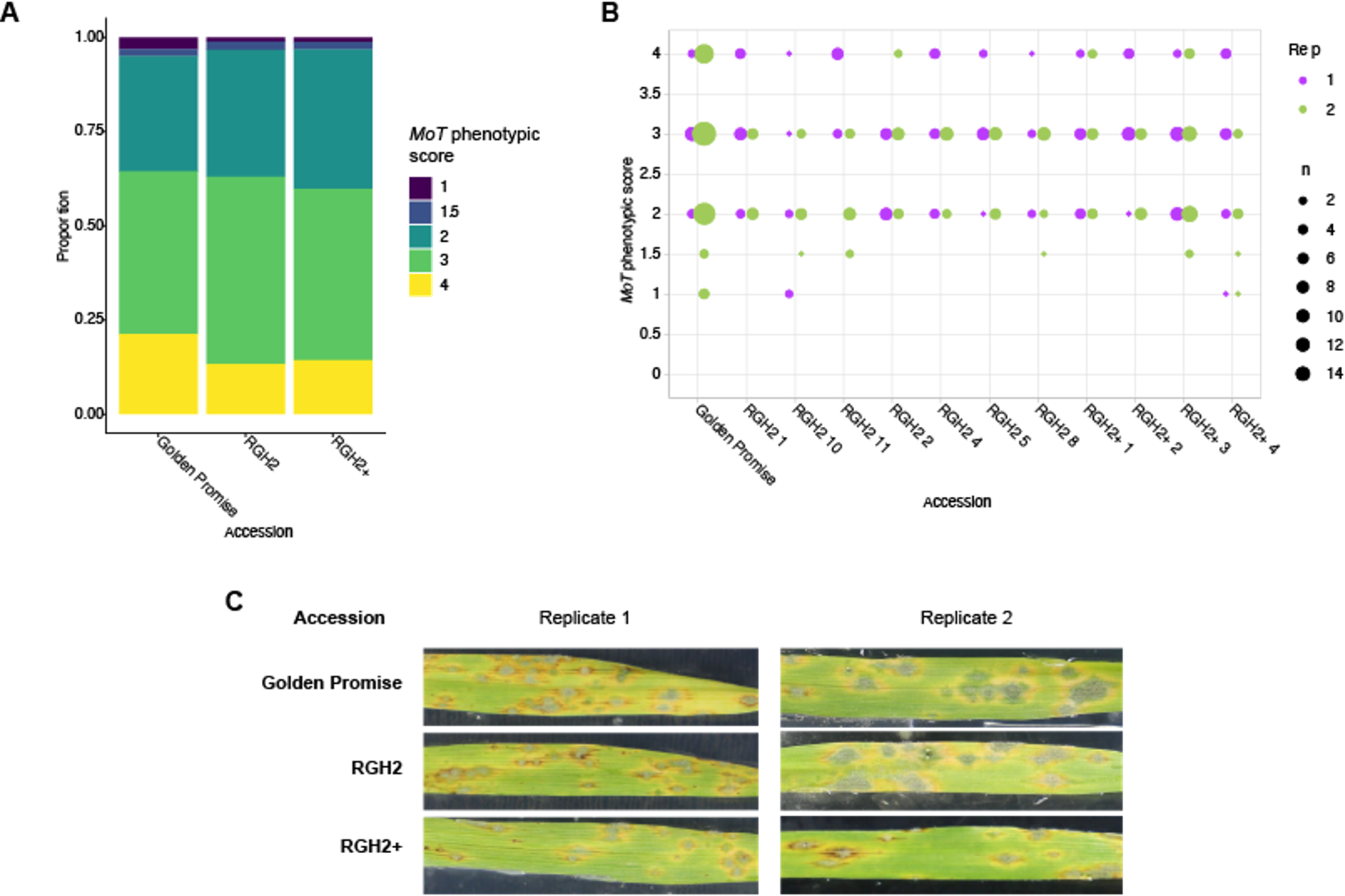

Supplement: koag168_Supplementary_Data [file koag168_supplementary_data.zip › Figure_S14.tiff]

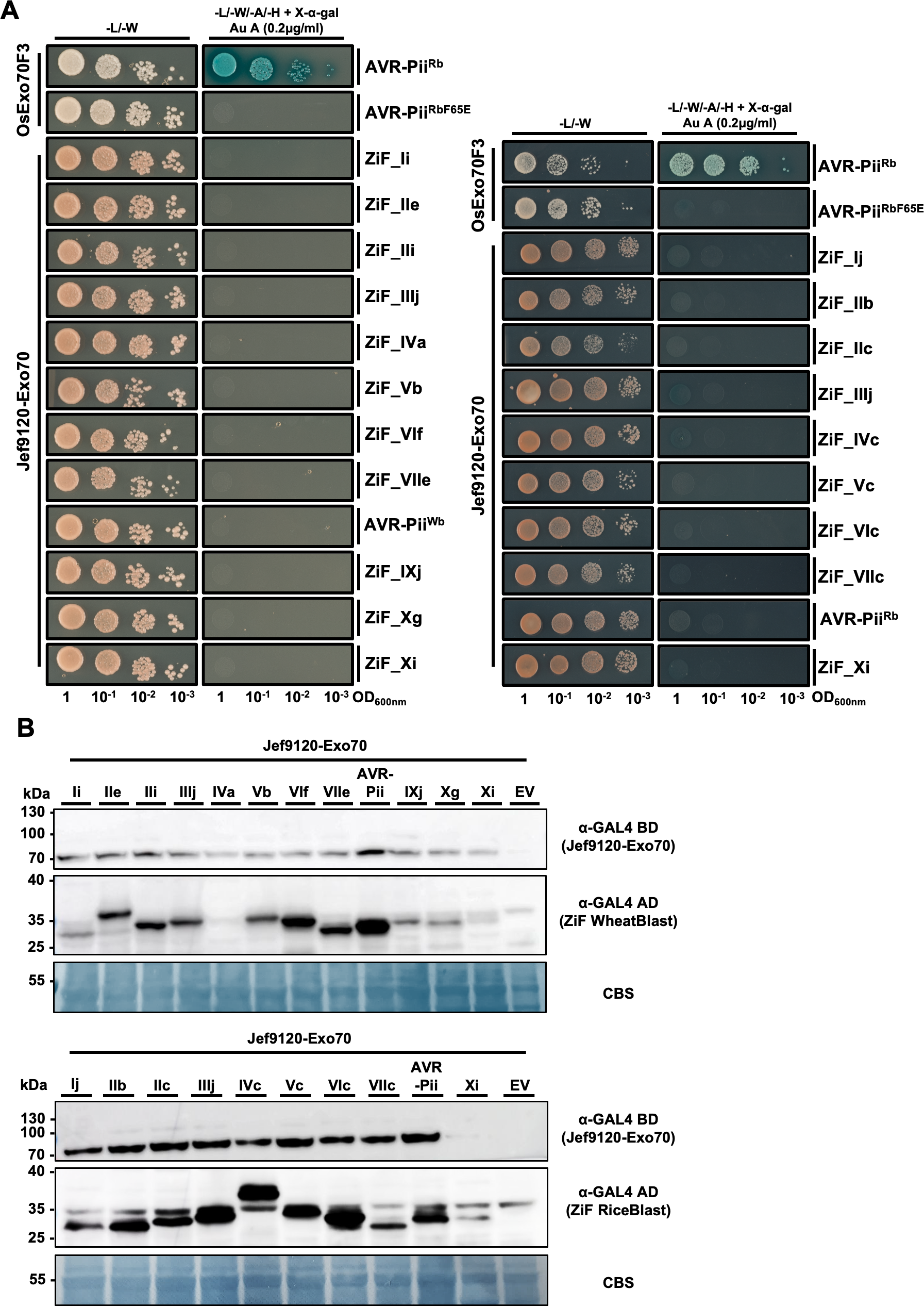

Supplement: koag168_Supplementary_Data [file koag168_supplementary_data.zip › Figure_S2.tiff]

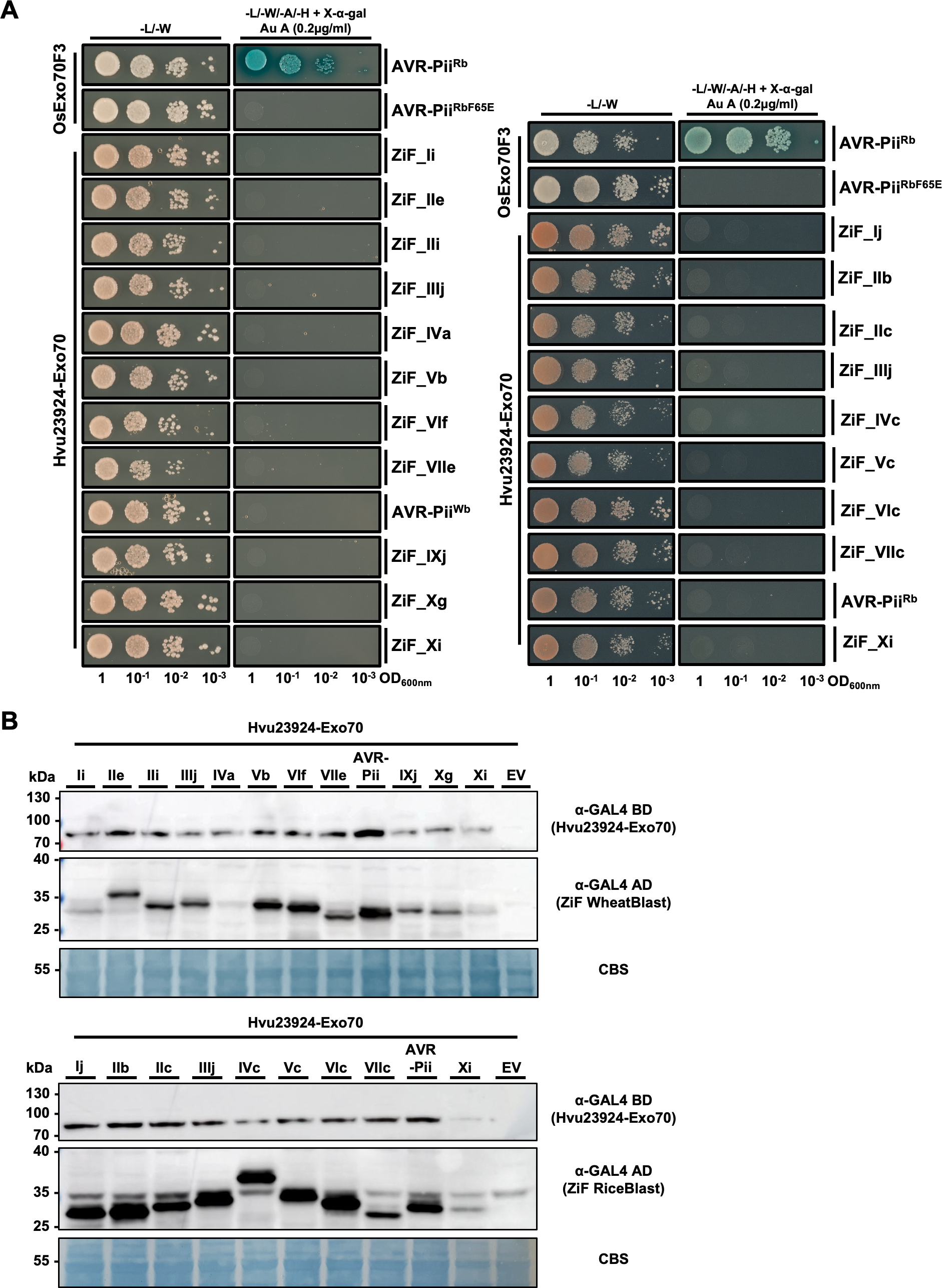

Supplement: koag168_Supplementary_Data [file koag168_supplementary_data.zip › Figure_S3.tiff]

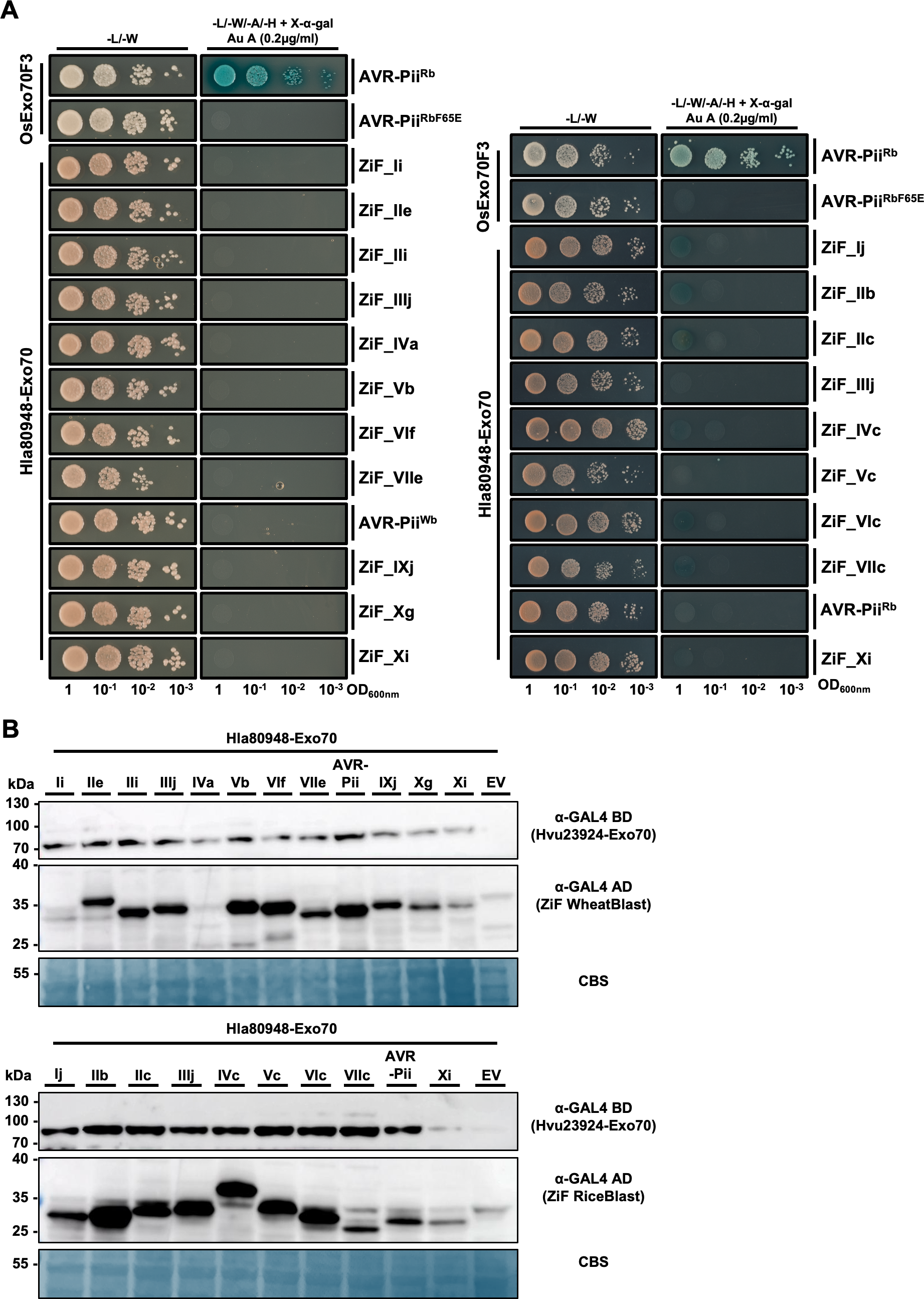

Supplement: koag168_Supplementary_Data [file koag168_supplementary_data.zip › Figure_S4.tiff]

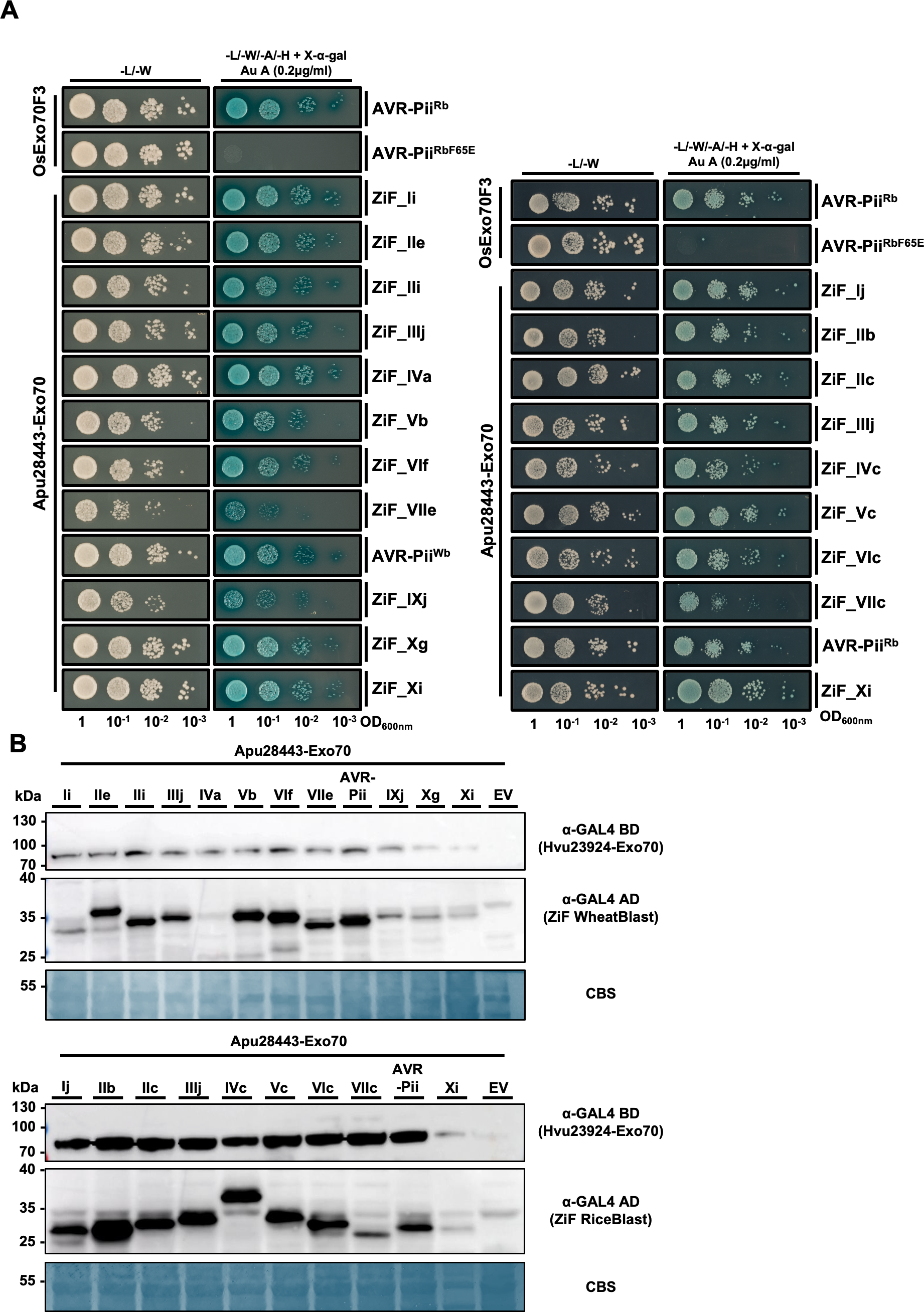

Supplement: koag168_Supplementary_Data [file koag168_supplementary_data.zip › Figure_S5.tiff]

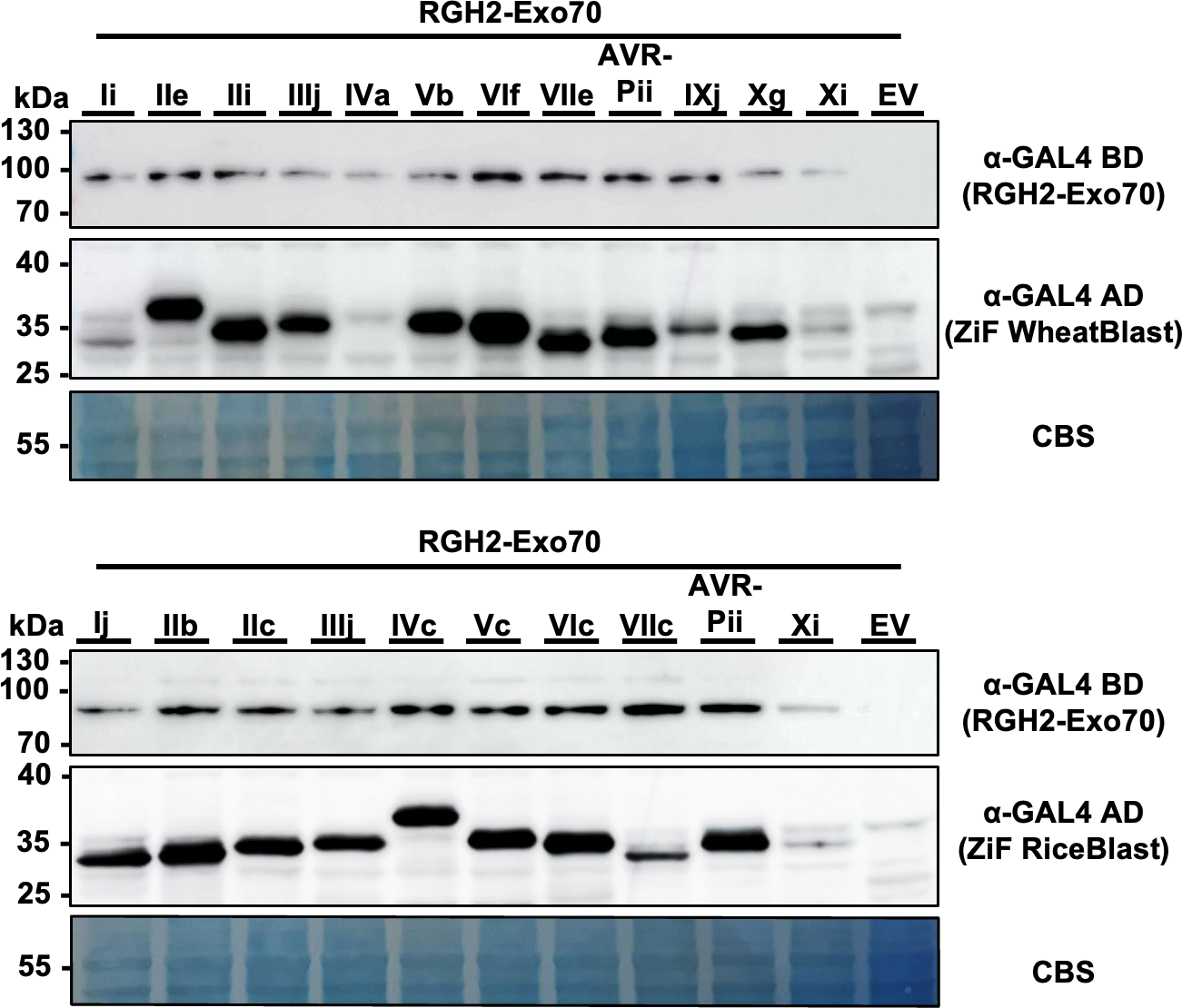

Supplement: koag168_Supplementary_Data [file koag168_supplementary_data.zip › Figure_S6.tiff]

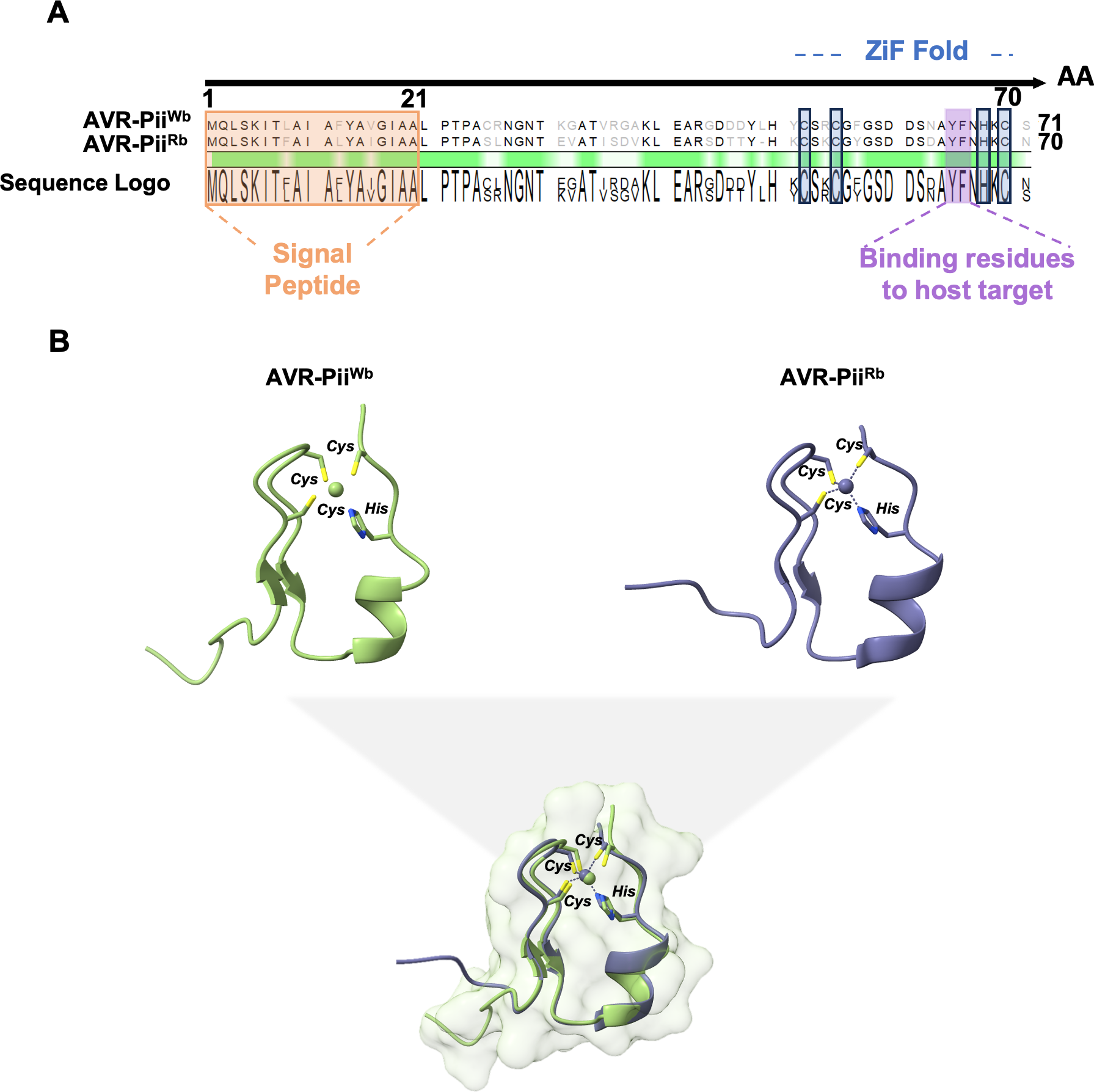

Supplement: koag168_Supplementary_Data [file koag168_supplementary_data.zip › Figure_S7.tiff]

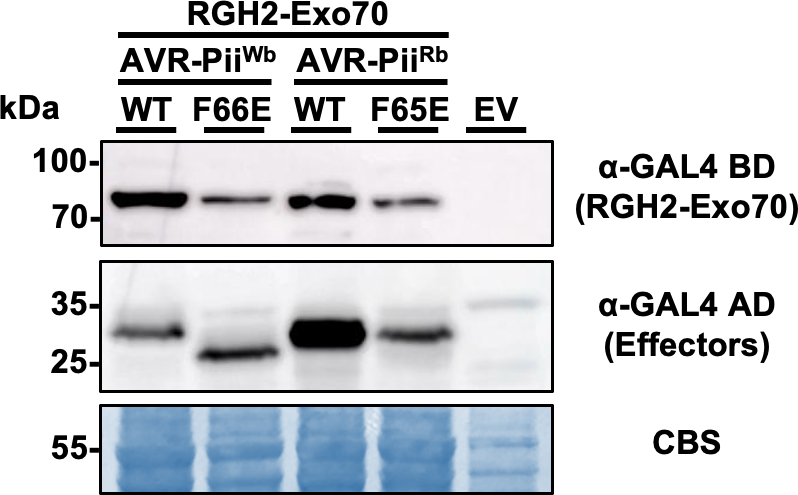

Supplement: koag168_Supplementary_Data [file koag168_supplementary_data.zip › Figure_S8.tiff]

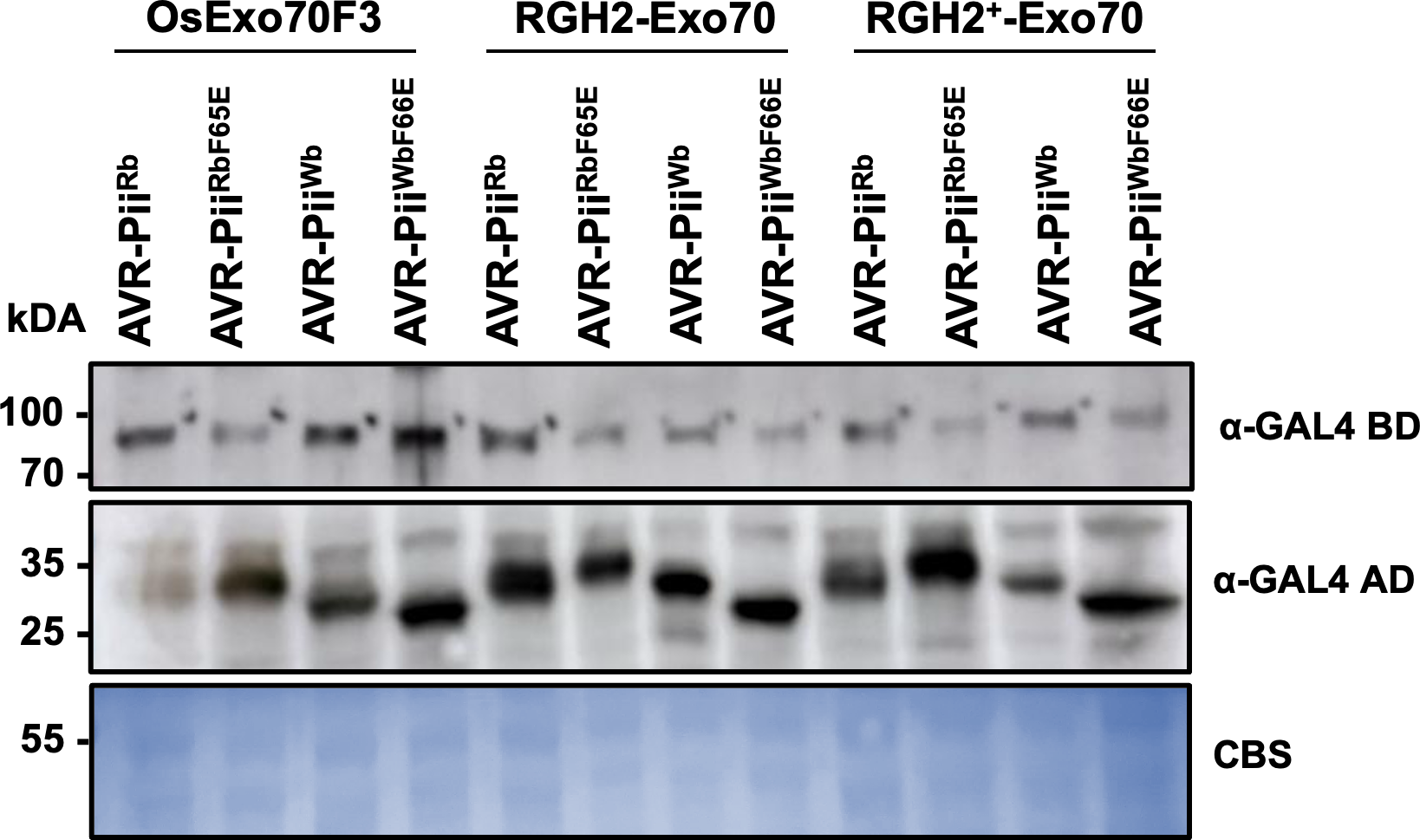

Supplement: koag168_Supplementary_Data [file koag168_supplementary_data.zip › Figure_S9.tiff]
